# Realigning the LIGHT signaling network to control dysregulated inflammation

**DOI:** 10.1084/jem.20220236

**Published:** 2022-05-23

**Authors:** Carl F. Ware, Michael Croft, Garry A. Neil

**Affiliations:** 1 Infectious and Inflammatory Diseases Center, Sanford Burnham Prebys Medical Discovery Institute, La Jolla, CA; 2 Division of Immune Regulation, La Jolla Institute for Immunology, La Jolla, CA; 3 Avalo Therapeutics, Inc., Wayne, PA

## Abstract

Advances in understanding the physiologic functions of the tumor necrosis factor superfamily (TNFSF) of ligands, receptors, and signaling networks are providing deeper insight into pathogenesis of infectious and autoimmune diseases and cancer. LIGHT (*TNFSF14*) has emerged as an important modulator of critical innate and adaptive immune responses. LIGHT and its signaling receptors, herpesvirus entry mediator (*TNFRSF14*), and lymphotoxin β receptor, form an immune regulatory network with two co-receptors of herpesvirus entry mediator, checkpoint inhibitor B and T lymphocyte attenuator, and CD160. Deciphering the fundamental features of this network reveals new understanding to guide therapeutic development. Accumulating evidence from infectious diseases points to the dysregulation of the LIGHT network as a disease-driving mechanism in autoimmune and inflammatory reactions in barrier organs, including coronavirus disease 2019 pneumonia and inflammatory bowel diseases. Recent clinical results warrant further investigation of the LIGHT regulatory network and application of target-modifying therapeutics for disease intervention.

## Teaser

The cytokine LIGHT (TNFSF14) has emerged as an important modulator of innate and adaptive immune responses. Accumulating basic and clinical evidence points to the dysregulation of the LIGHT network as a disease-driving mechanism and supports the application of target-modifying therapeutics for disease intervention.

## Introduction

The TNF superfamily (TNFSF) of cytokines provides critical communication pathways coordinating innate and adaptive immune responses. These cytokines initiate specific signaling pathways through a large superfamily of cognate cell surface receptors (TNFRSF) to create several immunoregulatory networks. The development of several efficacious therapeutics in autoimmune and inflammatory diseases emerged from understanding fundamental features of the TNFSF in immunity. The focus of this review centers on the TNFSF-related cytokine, LIGHT (lymphotoxin-like, exhibits inducible expression, and competes with HSV glycoprotein D for herpesvirus entry mediator [HVEM], a receptor expressed by T lymphocytes), encoded by the *TNFSF14* gene. LIGHT is intimately linked to several signaling pathways as a key component in a larger immunoregulatory network (Ward-Kavanagh et al., 2016). Importantly, the expression in immune effector cells and presence in inflamed tissues, including coronavirus disease 2019 (COVID-19), places LIGHT as a priority candidate for immunotherapy. The complexity of the ligand–receptor interactions of LIGHT and other members of the TNFSF family poses significant and ongoing challenges in defining their physiologic functions and their role in various disease conditions. However, accumulating data provide a reasonable blueprint to predict clinical indications in which to target LIGHT-mediated pathways.

LIGHT is structurally related to TNF, lymphotoxin (LT)-α, and LTβ (LTαβ) and discovered as a ligand for the TNFRSF member HVEM (*TNFRSF14*; Mauri et al., 1998). Interestingly, HVEM was originally identified as a receptor for the HSV virion glycoprotein D, which utilizes HVEM to infect activated T cells and appears to have evolved as an inhibitory modulator of LIGHT–HVEM signaling (Aschner and Herold, 2021). LIGHT engages a second signaling receptor, the LTβ receptor (LTβR), which modulates trafficking of lymphocytes and builds and maintains the architecture of lymphoid tissues into effective host defense systems. The trimeric structure of LIGHT with three receptor binding sites yields high avidity binding that promotes receptor clustering, which in turn initiates intracellular signaling pathways in receptor-bearing cells. The structural signature of TNFRSF is a cysteine-rich ectodomain forming a ladder-like molecule with a cytosolic domain activating the NF-κB family of transcription regulators (Ward-Kavanagh et al., 2016). LIGHT engagement of HVEM provides a costimulatory signal for T and B cell activation. HVEM signals via the cytoplasmic ubiquitin E3 ligase TRAF2/cIAP complex activating the NF-κB RelA transcriptome of inflammatory, proliferative, and survival genes important for immune function. In contrast, LTβR signaling initiates NF-κB RelB-dependent gene transcription, such as chemokines CxCL13, CCL19, and CCL21, involved in positioning T and B cells in germinal centers and formation of tertiary lymphoid structures creating de novo extra-lymphatic immune environments.

The cellular response outcomes depend in part on the expression patterns of LIGHT, HVEM, and LTβR, and the bioavailability of LIGHT. Naive T cells require cellular activation signals to induce the transcription and protein expression of LIGHT. Similar to other TNFSF members, the membrane form of LIGHT mediates responses between cells in direct contact. LIGHT is also shed into a soluble cytokine with potential systemic effects. Both the membrane and soluble forms of LIGHT induce signaling via HVEM or LTβR, typically measured by activation of NF-κB transcription factors. LIGHT is primarily expressed in inflammatory effector cells, including dendritic cells, natural killer (NK) cells, macrophages, neutrophils, innate lymphoid cells, NK T cells and activated effector, and CD4 and CD8 memory T cells, but not in naive T cells, regulatory T cells, or B cells (Heng and Painter, 2008; Savage et al., 2021), a pattern implicating its role in both acute inflammatory and adaptive immune responses. HVEM is expressed widely in all lymphocyte populations, including naive CD4 and CD8 T and B cell subsets and in some endothelial, epithelia, and fibroblastic reticular cells in barrier organs, a pattern suggesting LIGHT–HVEM is an important immunomodulatory mechanism. In contrast, LTβR expression is limited to stromal and myeloid cells, yet strikingly absent in all lymphocyte lineages. LTβR signaling in stromal cells serves to modify tissue microenvironments in lymph nodes and at sites of inflammation.

LIGHT and LTαβ differ in their roles in activating the LTβR. LTαβ expressed in fetal lymphoid tissue inducer cells is required for the development of secondary lymphoid organs (e.g., lymph nodes, Peyer’s patches), whereas the architectural integrity of lymph nodes requires B cell LTαβ expression. In contrast, innate and activated T effector cells expression of LIGHT can promote cellular infiltration into extra-lymphatic tissues via the LTβR.

Genetic or pharmacologic inhibitors of the LTαβ and LTβR pathway cause disorganization of the intricate architecture and loss of function in lymphoid organs, especially apparent in host defense, including failure to mount type I IFN (IFN-I) response to viral pathogens or form germinal centers for IgG class switch. Formation of tertiary lymphoid structures at sites of persistent inflammation is promoted by LIGHT–LTβR signaling. In mice engineered to constitutively express LIGHT in T cells, induction of systemic inflammation in the intestine, which resembles Crohn’s disease–like pathology, was observed (Shaikh et al., 2001; Wang et al., 2005) and associated with production of excessive Th1 cytokines by mucosal T cells. The results indicate that LIGHT functions as an important regulator of T cell activation and implicate LIGHT in inflammatory signaling pathways.

## LIGHT–HVEM–B and T lymphocyte attenuator (BTLA) signaling network

**Figure 1.**
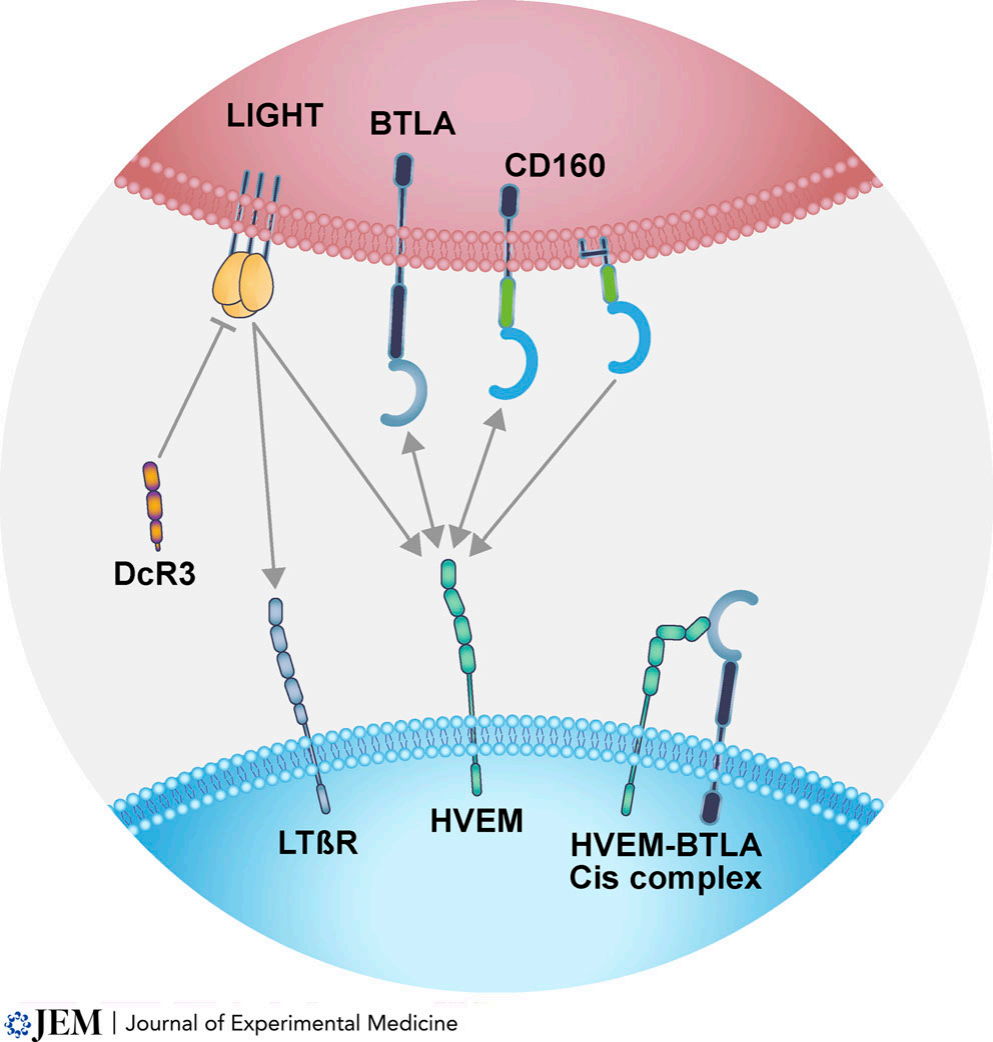
**LIGHT signaling network.** The arrows indicate the ligand–receptor interactions; single arrows indicate monodirectional; double-headed arrows indicate bidirectional signaling. LIGHT is a ligand for both HVEM and LTβR. DcR3 competitively inhibits LIGHT binding to LTβR and HVEM, blocking their signaling actions. LIGHT expression requires activation of T cells, whereas differentiated effector cells, such as neutrophils and NK cells, constitutively express LIGHT. HVEM also binds the two immunoglobulin superfamily members, BTLA and CD160. CD160 has two forms, as transmembrane and the dominant glycosphingolipid-linked form. In cells that coexpress HVEM, CD160, and BTLA, CD160 competes with BTLA for HVEM serving to downmodulate inhibitory BTLA signaling. As an example of bidirectional signaling, HVEM activates BTLA’s inhibitory pathway and reciprocally BTLA initiates HVEM’s activation of NF-κB transcription factors. Lymphocytes can coexpress HVEM, BTLA and CD160, forming complexes *in cis*. For example, naive T cells are initially restricted to BTLA and HVEM coexpression; however, following activation CD8 effector T cells coexpress all four proteins. The binding interactions among these proteins occur at intercellular contacts, such as T cell–dendritic cells during antigen recognition. Soluble LIGHT binds its receptors with high affinity, thus acting in a systemic fashion. In contrast, the relative low affinity of HVEM for BTLA and CD160 requires cell contact to activate signaling.

The discovery of LIGHT and its receptors, HVEM and LTβR, provided a glimpse into a larger network defined by co-receptors, shared ligands, and bidirectional signaling. HVEM also engages two members of the Ig superfamily, the inhibitory checkpoint receptor, BTLA, and CD160. The HVEM–BTLA and HVEM–CD160 pathways place HVEM as a central hub controlling both proinflammatory and inhibitory signaling (Fig. 1). Additionally, the shared ligand–receptor interactions of LIGHT include the commonality with LTαβ and the LTβR and TNFR1, TNFR2, and HVEM with LTα as the shared ligand. The circuits created by LIGHT link both HVEM and LTβR signaling pathways, promoting responses in both lymphocyte and stromal compartments (Ward-Kavanagh et al., 2016).

The consequences of HVEM–BTLA checkpoint include inhibitory action limiting the activation and proliferation of B and T cells and cytokine signaling. Inhibitory signaling by BTLA occurs through recruitment of the tyrosine phosphatases SHP1/2 limiting signaling by T and B cell antigen and cytokine receptors, thus restricting the intensity of inflammatory responses (Steinberg et al., 2011; Ward-Kavanagh et al., 2016). HVEM–BTLA engagement occurs in *trans*, allowing bidirectional signaling. T and B lymphocytes also coexpress HVEM with BTLA forming complexes in *cis* (Cheung et al., 2009a; Cheung et al., 2009b). The HVEM–BTLA cis complex is thought to maintain non-antigen activated T and B cells in a naive state. Structural analysis revealed BTLA and CD160 bind overlapping sites on HVEM but occupy distinct domains from LIGHT and LTα, creating variations in ligand–receptor complexes and consequences for signaling pathways (Carfi et al., 2001; Compaan et al., 2005; Liu et al., 2014; Liu et al., 2021). Innate effector cells such as NK cells and γδT cells coexpress HVEM, CD160, and BTLA with a high CD160:BTLA ratio. The absence of an inhibitory signaling domain in CD160 and competitive binding can block access of BTLA to HVEM, thus limiting BTLA inhibitory signaling, a feature of CD160–HVEM interaction suggested to maintain effector cells poised for rapid response to pathogens (Šedý et al., 2013).

The bioavailability of LIGHT is regulated by another TNFRSF member, decoy receptor 3 (DcR3; *TNFRSF6b*), a soluble protein that binds and sequesters LIGHT with equivalent affinity to HVEM and LTβR. The structural analysis of LIGHT–DcR3 reveals a 3:1 receptor: ligand complex typical of the TNFSF. DcR3 also binds TL1A (*TNFSF15*) and FAS ligand (*FASLG*), providing an important control mechanism for these cytokines (Liu et al., 2014). The DcR3 gene is present in all mammalian species except for *Murinae*, an important caveat in assessing LIGHT regulation in mouse models. DcR3 circulates in plasma and is bound in tissues through cell surface glycosaminoglycans, thus limiting the systemic bioavailability of LIGHT in tissue microenvironments. Epithelial cells and innate T cells secrete DcR3 in response to inflammatory signals during sepsis (Kim et al., 2005; Kim et al., 2012). The levels of LIGHT in severe COVID-19 may exceed the capacity of DcR3, suggesting a mechanism underlying cytokine dysregulation syndrome (Perlin et al., 2020). The dynamics of DcR3–LIGHT interaction and consequences of dysregulation of this mechanism as a source of pathology require further investigation.

## Role of LIGHT in disease pathogenesis

### LIGHT in host defense systems

Pathogens have illuminated the importance of the LIGHT network in innate and adaptive immune responses. LIGHT–HVEM–BTLA pathways are directly targeted by several viruses; particularly informative are herpesviruses, which require both innate and adaptive defenses to control their characteristic persistent and latent life cycle. For example, human CMV, a β-herpesvirus, encodes a viral mimic of HVEM (UL144 ORF) that functions as an agonist specific for BTLA (Bitra et al., 2019; Šedý et al., 2017), and variants are associated with serious congenital CMV infection (Waters et al., 2010). The requirement for LTβR signaling in initiating the innate IFN-I response was demonstrated in human and mouse infection models with CMV and other viral pathogens (Gommerman et al., 2014; Koroleva et al., 2018). The rapid IFN-I response depends on the LTβR-mediated differentiation of stromal cells and marginal zone macrophages as a part of the homeostatic organization of lymphoid organs.

The LIGHT–HVEM pathways are intimately involved in host mechanisms needed for innate and adaptive defenses to limit the immune evasion strategies of viral pathogens. Mice genetically deficient in components of HVEM’s pathways show either increased susceptibility or attenuated pathogenesis in response to different viral pathogens. For example, the HSV envelope glycoprotein D effectively disrupts LIGHT–HVEM signaling suppressing the production of antigen-specific IgG2a/c, the antibody subclass required for virus clearing via antibody-dependent cytotoxicity and phagocytosis (Burn Aschner et al., 2020). LIGHT–HVEM signaling participates in the effector phase of antibody-dependent cellular cytotoxicity and may serve as tissue destructive mechanism in autoimmune diseases. Other studies indicate LIGHT, BTLA, and CD160 can act as equivalent activating ligands for HVEM in the immunopathogenesis of ocular HSV-1 infection (Park et al., 2020). Surprisingly, attenuated ocular pathology was observed in mice deficient in HVEM and with a dual deficiency in BTLA LIGHT or CD160 LIGHT, but no differences were seen in mice with single-gene deficiency in either LIGHT, BTLA, or CD160 or wild-type mice (Park et al., 2020). The double knockout mice showed reduced levels of infiltrating leukocytes and decreased IFN-γ producing CD4^+^ T cells. These results may represent the potential redundant aspects of LIGHT, BTLA, and CD160 in activating HVEM. In another viral model, the CD8^+^ T cell response against vaccinia (*Orthopoxvirus*) revealed that protective immunity was dependent on the interaction of HVEM and BTLA in *trans* configuration with HVEM expressed in CD8^+^ T-cells and BTLA in dendritic cells (Flynn et al., 2013). Moreover, HVEM but not BTLA was necessary for the continued survival of virus-specific effector CD8^+^ T cells and optimal generation of memory (Flynn et al., 2013). Additional results showed that LIGHT provides a critical signal in the production of lung-resident memory CD8 T cells following an acute respiratory viral infection (Desai et al., 2018). In this setting, very few LIGHT-deficient CD8^+^ T cells survived, severely compromising the memory compartment, although activation, proliferation, functionality, and trafficking appeared normal, perhaps accounted for by an unknown homeostatic survival signaling mechanism lacking in the microenvironment of the lung resident memory T cells.

The host–pathogen infection models demonstrate unique and shared ligands and bidirectional pathways of the HVEM and LTβR pathways. Importantly, these pathways may be operative in non-infectious autoimmune diseases, such that targeting this network may halt tissue damage by inhibiting survival of memory T cells or dismantling tertiary lymphoid clusters.

### LIGHT in inflammatory diseases

Emerging evidence has identified LIGHT in the pathogenesis of inflammatory and autoimmune disorders (Table 1). The clinical relevance of LIGHT was demonstrated in a study conducted in patients hospitalized with COVID-19 (Perlin et al., 2020). In this study, hospitalized patients had significantly higher LIGHT levels than healthy age- and gender-matched controls. The elevated serum levels of LIGHT were within the receptor activation concentrations and likely exceeded the sequestering action of DcR3 (Qu et al., 2022). Moreover, in hospitalized patients over the age of 60, who had a mortality rate of 82%, LIGHT plasma levels were significantly higher in those who died than in those who survived. In another study that utilized a systems approach to assess immunity in mild versus severe COVID-19, LIGHT was among three serum proteins significantly enhanced in COVID-19 disease and strongly correlated with clinical severity (Arunachalam et al., 2020). Additional reports confirmed significantly increased plasma levels of LIGHT that correlated with COVID-19 severity (Haljasmägi et al., 2020; Tan et al., 2021). Interestingly, infection with influenza or respiratory syncytial virus infections showed no elevation in LIGHT (Arunachalam et al., 2020). In that study, the increase in LIGHT levels correlated with elevated bacterial products suggesting septic conditions may contribute to increased LIGHT in COVID-19 (Arunachalam et al., 2020) and in non–COVID-19 sepsis (Qu et al., 2022).

**Table 1.** LIGHT in disease and clinical applications

Disease	LIGHT function and evidence
COVID-19	LIGHT expression as a prognostic biomarker in COVID-19 (Arunachalam et al., 2020; Perlin et al., 2020; Tan et al., 2021)
IBD	Reduced intestinal inflammation by LIGHT neutralization in a colitis model (Jungbeck et al., 2009)
Asthma, IPF, SSc	LIGHT drives fibrosis and tissue remodeling in the lung (Doherty et al., 2011; Herro et al., 2015)
Atopic dermatitis, scleroderma	LIGHT induces changes in keratinocytes and promotes epidermal and dermal thickening (Herro et al., 2015, 2018)
Metabolic diseases	Elevated LIGHT level in patients with type 2 diabetes (Halvorsen et al., 2016) LIGHT-mediated inflammation implicated in nonalcoholic fatty liver disease (Herrero-Cervera et al., 2019)
Postmenopausal osteoporosis	Antagonizing LIGHT could be therapeutically beneficial in patients with postmenopausal osteoporosis (Brunetti et al., 2020)
Cancer	LIGHT was found to play a role in antitumor immunity (Holmes et al., 2014) LTβR signaling implicated in tumor metastasis by inducing anti-tumor effector cells (Lu and Browning, 2014)
RA	Lymphotoxin/LIGHT axis decreases the IFN signature in patients’ blood cells (Bienkowska et al., 2014)

Collectively, these findings suggest that LIGHT may serve as a prognostic biomarker for patients with COVID-19; however, further clinical validation is needed. Inflammatory cytokine networks have also been implicated in pediatric inflammatory multisystem syndrome pathogenesis associated with COVID-19 in children (Gruber et al., 2020).

In summary, LIGHT is emerging as a key player in the pathogenesis of COVID-19 and may represent a contributing factor in inflammatory cytokine dysregulation syndrome (Channappanavar and Perlman, 2017). It is important to note that the time-to-processing protocols with human blood can affect quantification of molecular and cellular aspects of the immune system (Savage et al., 2021). The level of LIGHT is stable for 4–6 h in human blood at room temperature but increases approximately twofold over 18 h, not unlike multiple cytokines in this study. The time to process becomes an important method to report in published articles.

### Gastrointestinal inflammation

**Figure 2.**
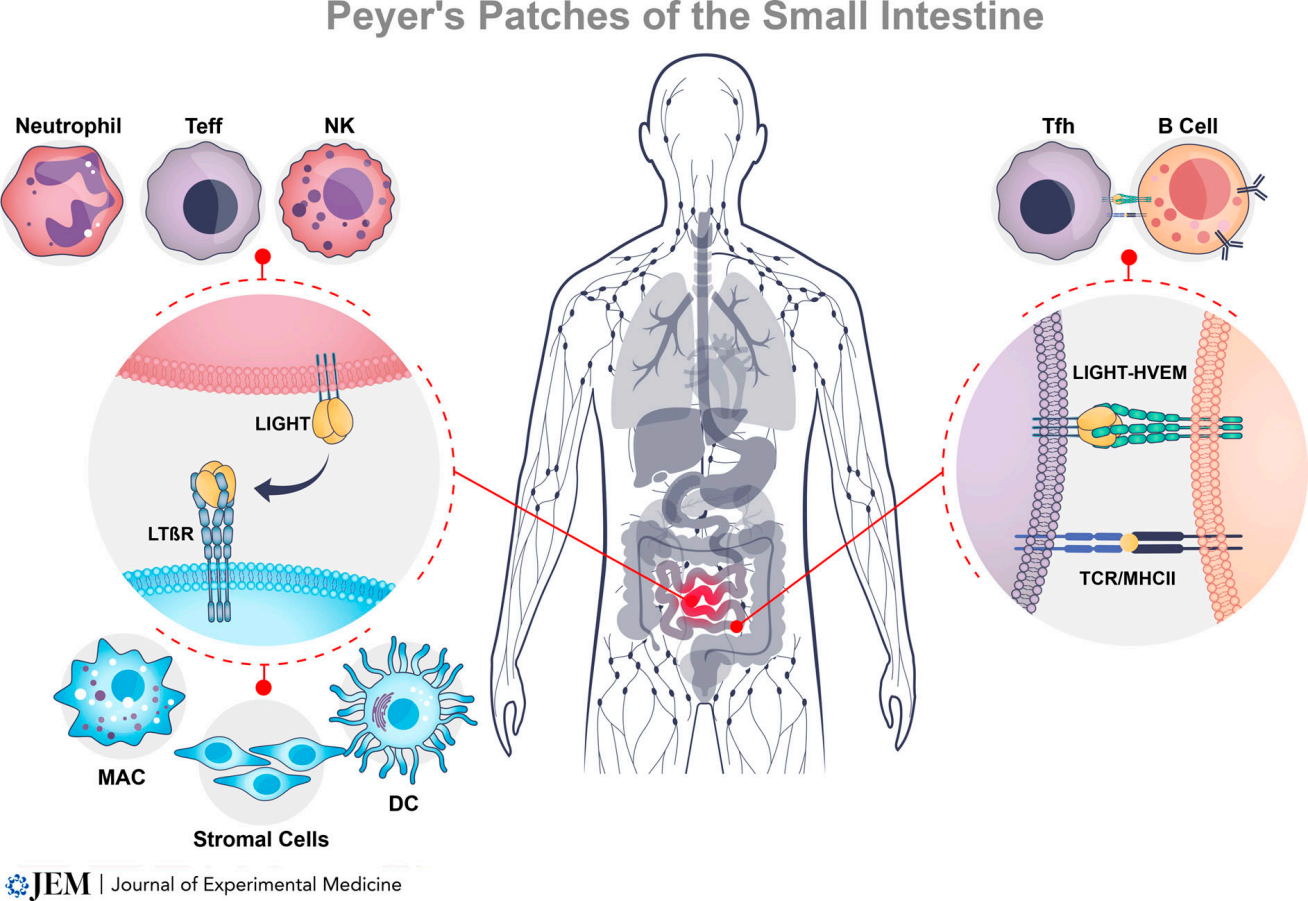
**LIGHT in intestinal inflammation.** The physiologic function of the LTβR pathway maintains the organization of secondary lymphoid organs (Peyer’s patches, spleen, lymph nodes) and also plays a key role in formation of tertiary lymphoid structures at sites of persistent inflammation. Innate pathogen-sensing receptors stimulate neutrophil and NK cell secretion of LIGHT at sites of inflammation. LIGHT activates LTβR to differentiate stromal cells into an immune niche favoring activation of antigen-presenting macrophages (MAC) and dendritic cells (DC) to recruit T and B cells. LIGHT activates the HVEM pathway to promote rapid recall responses and formation of germinal centers where T follicular helper cells (Tfh) promote antibody production by B cells. In patients with IBD, persistent intestinal inflammation may occur from a reinforcing cycle of LIGHT secretion by innate effector cells following disruption in the integrity of the mucosal epithelial barrier and re-exposure of T cells to self-antigens.

A number of studies in mouse models and human clinical observations have linked LIGHT to inflammatory bowel disease (IBD). In initial studies in models of colitis, T cell transfer into SCID mice or bone marrow chimeras with Tgε26 mice revealed that treatment with LTβR-Fc fusion protein, which neutralizes both LTαβ and LIGHT, reduced gut inflammation (Mackay et al., 1998), with subsequent experiments replicating those results in a model driven by a chemical hapten (An et al., 2005). The aforementioned findings were directly linked with LIGHT when constitutively expressed LIGHT in T cells exhibited a number of features of Crohn’s disease–like pathology, including immune infiltration of the small intestine and colon, loss of goblet cells, crypt hyperplasia, villus atrophy, and IgA nephropathy (Shaikh et al., 2001; Wang et al., 2001; Wang and Fu, 2004). These inflammatory effects were dependent on both HVEM and LTβR with responding pathogenic T cells and gut epithelial cells (Wang et al., 2005; Schwarz et al., 2007; Wang et al., 2010; Schaer et al., 2011). Correspondingly, LIGHT was found constitutively expressed on the surface of human lamina propria T cells, as well as NK cells, in the small intestine of IBD patients, and was upregulated to a greater extent in these patients’ T cells compared to normal T cells upon further activation (Cohavy et al., 2004; Cohavy et al., 2005; Fig. 2). LIGHT mRNA was also more strongly expressed in inflamed vs. non-inflamed intestines from patients with colitis (Cohavy et al., 2005) or in patients with active vs. inactive Crohn’s disease (Wang et al., 2005).

LIGHT also appears capable of mediating regulatory activities in some situations. In models of dextran sodium sulfate colitis, the extent of gut inflammation may be more dependent on the balance of protective vs. pathogenic innate immune cells. LIGHT signaling through LTβR, rather than HVEM, attenuated dextran sodium sulfate–induced colitis, with *Light*^−*/*−^ and *Ltbr*^−/−^ mice exhibiting a more severe pathology. However, adverse effects associated with the loss of LIGHT were reversed with the deletion of both *Ltβ* and *Tnfsf14*, but not deletion of both *Ltβ* and *Ltβr* (Krause et al., 2014; Steinberg et al., 2008; Giles et al., 2018). This paradoxical phenotype was recently shown to be due to an activity of LTβR signaling in neutrophils that suppressed production of reactive oxygen species and might have altered the balance of gut bacteria that could contribute to overall inflammation (Riffelmacher et al., 2021). However, whether this protective activity of LIGHT in the gut is relevant for subsets of IBD patients is unknown. Pertinent to this question is the analysis of the human colonic mesenchyme using single-cell RNA sequencing in patients with ulcerative colitis (Kinchen et al., 2018). The results yielded the unexpected presence of LIGHT and HVEM expressed in a novel population of proliferating fibroblastic reticular cells with a proinflammatory signature. In human WNT1 proto-oncogene–dependent intestinal organoid model, LIGHT or IL-6 suppressed expression of *LGR5*, *OLFM4*, *AXIN2*, *ALDHA1*, *CDX2*, and *NOTCH1* genes associated with epithelial stem cell proliferation; however, WNT withdrawal from the organoid cultures showed LIGHT specifically increased the expression of *LGR*5 and *AXIN2,* including the damage-responsive stem cell marker *MSI1*. Together, these data indicate that LIGHT and other inflammatory mediators may provide signals that aid in maintaining and regenerating the intestinal epithelium.

In addition to IBD, more recent results illustrated the potential pathogenic activity of LIGHT in eosinophilic esophagitis (EoE). LIGHT was initially visualized in esophagus tissue in a limited study of patients with EoE (Zhang et al., 2013). This result was expanded with single-cell RNA sequencing and further tissue staining, finding LIGHT more strongly expressed in all T cell subsets that accumulated in the epithelium and lamina propria of EoE patients, including the Th2 subset that is thought to be a main disease driver (Manresa et al., 2020). EoE patients often exhibit esophageal rigidity and lumen narrowing, thought to be due to epithelial hyperplasia and collagen deposition, the latter associated with an accumulation of fibroblasts displaying inflammatory and fibrotic phenotypes. Both HVEM and LTβR are expressed in esophagus fibroblasts, with HVEM more strongly expressed in cells from EoE patients. Signals from LIGHT drove expression of a number of inflammatory proteins in these fibroblasts via both receptors, interestingly coinciding with proteins expressed in inflammatory fibroblasts found in patients with IBD. Further linking LIGHT to esophageal inflammation, LIGHT-activated esophageal fibroblasts adhered to and clustered with eosinophils, a feature found in esophagus epithelium and lamina propria in EoE patients (Manresa et al., 2020; Manresa et al., 2022).

### Role of LIGHT in lung inflammation

LIGHT may also be central to several lung inflammatory diseases. LIGHT expression was first found increased in T cells from lung lavages of patients with systemic sclerosis (SSc) with pulmonary fibrosis (Luzina et al., 2003). Higher sputum levels of soluble LIGHT, or cells expressing LIGHT, have also been found to correlate with lowered lung function in asthmatics (Hastie et al., 2010; Hirano et al., 2021) and elevated mRNA for LIGHT in sputum cells clustered with several other markers in subsets of asthmatics with most severe disease (Frøssing et al., 2022). Connecting these observations to a likely role for LIGHT in driving lung tissue remodeling that is a feature of severe SSc and asthma and idiopathic pulmonary fibrosis (IPF), LIGHT-deficient mice, and mice therapeutically treated with LTβR-Fc, exhibited markedly less collagen deposition, bronchial smooth muscle mass, and airway hyperreactivity in T cell–dependent models of allergen-driven asthma and bleomycin-driven SSc/IPF (Doherty et al., 2011; Herro et al., 2015). Concordantly, injection of recombinant LIGHT alone into the lungs of mice induced these features of lung tissue remodeling (Doherty et al., 2011; Herro et al., 2015). Similarly, rhinovirus infection via the lungs promoted LIGHT expression and resulted in LIGHT-dependent tissue remodeling of the airways, which further exacerbated that induced by allergen (Mehta et al., 2018). Additionally, a recent study found that LIGHT, LTαβ, and signaling pathways associated with LTβR were upregulated in lung biopsies from patients with smoking-associated chronic obstructive pulmonary disease. Correspondingly, blockade of LIGHT with decoy LTβR-Fc suppressed lung fibrosis and inflammation induced by chronic exposure to cigarette smoke in a mouse model of chronic obstructive pulmonary disease (Conlon et al., 2020).

LIGHT has several reported activities that could explain its importance in inflammatory processes. LIGHT can regulate through HVEM the survival of memory T cells that control several of these diseases (Soroosh et al., 2011). Additionally, LIGHT can promote lung macrophages to produce TGF-β, the cytokine involved in driving fibroblast differentiation into collagen-producing myofibroblasts (Doherty et al., 2011). It also activates HVEM-mediated production of IL-13, a cytokine that can act on several structural cells in the lungs and promote tissue inflammation, either from eosinophils (Doherty et al., 2011) or mast cells (Sibilano et al., 2016). With dependence on LTβR, LIGHT also exhibits direct activity in lung epithelial cells (Mikami et al., 2012; da Silva Antunes et al., 2015; Hung et al., 2015; Conlon et al., 2020) and lung fibroblasts (da Silva Antunes et al., 2018), inducing a variety of cytokines, chemokines, fibrotic factors, and/or proliferation. Therefore, if LIGHT is upregulated in the lungs, it has the potential to be a primary driver of dysfunction in multiple diseases through directly or indirectly controlling inflammation of the tissue’s cells. The origin of LIGHT in the lungs is not clear but could primarily be T cells (Luzina et al., 2003; Soroosh et al., 2011) as in other tissues, although expression of LIGHT has been found in lung eosinophils, neutrophils, and epithelial cells (Esnault et al., 2013; Mehta et al., 2018; Hirano et al., 2021), suggesting additional sources, similar to fibroblastic reticular cells being found to be a source of LIGHT in the intestine (Kinchen et al., 2018).

### Role of LIGHT in skin inflammation

Skin inflammatory diseases like scleroderma and atopic dermatitis exhibit features in common with lung and gastrointestinal tract diseases, including extensive hyperplasia of epithelial cells and accumulation of collagen-producing inflammatory fibroblasts. Increased levels of soluble LIGHT have been found in the circulation in atopic dermatitis or scleroderma, in part correlating with disease severity and several other biomarkers (Kotani et al., 2012; Gindzienska-Sieskiewicz et al., 2019), and increased levels in scleroderma skin lesions (Gindzienska-Sieskiewicz et al., 2019; Tsoi et al., 2019). LIGHT-deficient mice displayed strongly reduced epidermal thickening, with reduced collagen deposition in the dermis, in models of bleomycin-induced scleroderma and allergen-induced atopic dermatitis, along with lower expression of other markers of skin inflammation (Herro et al., 2015; Herro et al., 2018). Correspondingly, injection of recombinant LIGHT alone into the skin of mice drove features of both diseases (Herro et al., 2015; Herro et al., 2018). As before, several cell types might be relevant targets of LIGHT, but skin tissue cells are likely to be central to its activity. Epidermal keratinocytes and dermal fibroblasts express both HVEM and LTβR, and LIGHT can induce several effects in these cells, including hyperplasia, and inflammatory molecules such as thymic stromal lymphopoietin and periostin (Herro et al., 2015; Herro et al., 2018). Showing the direct requirement for LIGHT signaling in keratinocytes for disease, mice with conditional deletion of HVEM in these cells were protected from developing allergen-induced atopic dermatitis similar to LIGHT-deficient mice (Herro et al., 2018). Again, the source of LIGHT in the skin is not known, but T cells, eosinophils, neutrophils, fibroblasts, and keratinocytes are all possibilities.

### LIGHT-regulated metabolism

The basis for metabolic disturbance may lie in the role LIGHT–LTβR signaling has in directly affecting adipocyte differentiation. Several studies indicate that LTβR provides an NF-κB–mediated inhibitory effect controlling adipocyte precursor cells diverging their fate to lymphoid organ stromal cells, thus suppressing adipogenesis in both white and brown fat (Bénézech et al., 2012; Kou et al., 2019; Kou et al., 2021). The differentiating effect involved inhibition of the expression of the major adipogenic factors PPARγ and CEBPα. The divergence toward lymphoid stromal cell differentiation through LIGHT–LTβR signaling may initiate formation of tertiary lymphoid structures and provide an energy source for metabolic changes required for immune responses.

Clinical observations showed serum LIGHT concentrations significantly increased in morbid obesity and type 2 diabetes patients showing a positive correlation with fat mass, body mass index, glycated hemoglobin and fasting triglycerides, and negatively with high-density lipoprotein cholesterol (Bassols et al., 2010). Mouse models of nonalcoholic fatty liver disease–associated type 2 diabetes have not provided clear results using LIGHT-deficient mice (Herrero-Cervera et al., 2019; Saunders et al., 2018), highlighting the complexity of the LIGHT network in the design of LIGHT-modulating therapeutics.

## Targeting LIGHT in inflammation

Preclinical and clinical data strongly suggest that LIGHT and its receptors are potential targets for biologics that antagonize or activate these pathways. The recognition of the molecular and pharmacologic heterogeneity present in each clinically defined autoimmune disease has revealed the limitation of current therapies. For instance, TNF inhibitors, both antibody- and receptor-Ig–based biologics, have strong disease-attenuating efficacy in rheumatoid arthritis (RA), psoriasis, and IBD, with a clinical response limited to 30–40% of subjects. The first therapeutic directed to the LTαβ–LIGHT network, baminercept, an LTβR-Ig fusion protein that neutralizes both LIGHT and LTαβ, was tested in patients with RA (Bienkowska et al., 2014). A subset of RA patients in that study had circulating lymphocytes expressing an IFN-I–induced gene pattern (Barrat et al., 2019). The majority of RA patients did not respond clinically to baminercept; however, a subset of patients with the IFN-I signature showed a significant decrease in this signature, changes in associated biomarkers, and a trend toward clinical benefit; however, the power to demonstrate clinical significance was limited by the small number of IFN-I+ patients. Importantly, baminercept provided the first clinical evidence linking the LTαβ/LIGHT network to the IFN-I system, originally predicted from the fundamental infectious disease models. Recent approval of IFN receptor antagonist (anifrolumab-fnia) for lupus indicates the importance of IFN-I signaling in certain autoimmune disease processes. In Sjogren’s syndrome, the efficacy of baminercept was not significant (St Clair et al., 2018). In addition, the pateclizumab blocking the ability of LTα and LTαβ to activate TNFR1, TNFR2, and LTβR did not improve clinical outcome in an RA trial (Kennedy et al., 2014). Multiple reasons may account for the lack of efficacy of these biologics in RA or Sjogren’s disease including both drug mechanism and disease characteristics. The complex molecular mechanisms of both baminercept and pateclizumab preclude assigning a definitive answer to their roles in disease processes. Together, these results reinforce the complexity of autoimmune disease based on pharmacologic definitions.

Specific targeting of the LIGHT network may yield benefits for patients unresponsive to current therapeutics in other inflammatory conditions. The antagonist IL-4Rα mAb (dupilumab) approved for atopic dermatitis and asthma (Thibodeaux et al., 2019) primarily blocks the actions of IL-13 on epithelial cells, smooth muscle cells, and fibroblasts. Evidence demonstrating LIGHT induces IL-13 and integrates IL-13 signals (Doherty et al., 2011; Herro et al., 2015; da Silva Antunes et al., 2018; Herro et al., 2018) suggests patients who respond to IL-4Rα blockers will also respond to LIGHT antagonists. IL-4Rα antibodies are only effective in subsets of asthmatics and atopic dermatitis patients, and so a similar issue remains as to whether antibodies that block LIGHT will exert activity in those unresponsive subjects. Current therapeutics are limited in other inflammatory and fibrotic diseases, such as IPF and SSc/scleroderma; thus, interrogation of LIGHT, HVEM, and/or LTβR biomarkers will undoubtedly be important in identifying appropriate patients.

Human neutralizing mAb (IgG4) against LIGHT has been developed with clinical trials underway. AVTX002 (developed by Kyowa Kirin) neutralizes both soluble and membrane LIGHT (Zhang et al., 2017). AVTX-002 exhibited excellent pharmacokinetic/pharmacodynamic properties and safety profile in phase 1a studies of healthy patients (Zhang et al., 2017).

The progression to severe COVID-19 is associated with dysregulated immune response, which can result in cytokine-release syndrome and acute respiratory distress syndrome (ARDS). The urgency of COVID-19 spawned several clinical trials with anti-cytokine therapies in COVID-19. A recently completed phase II study of AVTX002 (aka CERC002) in patients with severe COVID-19 pneumonia revealed a significant protective effect (Perlin et al., 2022). This proof-of-concept trial enrolled hospitalized adults (*n* = 83, double blind) with COVID-19–associated pneumonia and mild to moderate ARDS. AVTX was administered with standard of care therapy (systemic corticosteroids or remdesivir). Plasma LIGHT was rapidly cleared by the antibody and associated with a higher proportion of patients that remained alive and free of respiratory failure (day 28) after receiving CERC-002 vs. placebo (P = 0.044). Patients >60 yr of age showed a pronounced effect with LIGHT neutralization. These results provide evidence for a direct link between LIGHT and disease pathogenesis.

In comparison with anti-LIGHT, analysis of the Cochrane Collaboration COVID-19 database (Ghosn et al., 2021) indicated treatment with antagonist IL-6 receptor mAb (tocilizumab) showed little or no increase in the clinical improvement at D28 but did reduce mortality. Similar disappointing results were seen with TNF inhibitors (Fakharian et al., 2021). Interestingly, neither TNF nor IL-6 blockade increased adverse effects, suggesting these cytokines may not serve critical pathways for host defense to COVID-19. Unfortunately, the datasets lacked sufficient biomarkers that limited identifying subsets of responding patients.

The positive results in COVID-19 ARDS provide the rationale to target inflammation in barrier tissues where LIGHT blockade may provide benefit. To this point, phase 2 trials include treatment of patients with non-eosinophilic asthma (National Library of Medicine, 2022a; NCT05288504) or moderate to severe Crohn’s disease who previously failed anti-TNF treatment (National Library of Medicine, 2021; NCT03169894). Another anti-LIGHT mAb CBS001 (Capella Bioscience) is directed at membrane LIGHT to target fibrosis and is currently in development for IPF. The results from these trials should provide additional insight into the clinical relevance of the LIGHT network.

Another approach targeting the broader LIGHT network in autoimmune diseases is directed at engaging the inhibitory signaling activity of BTLA. Preclinical studies support the concept that BTLA agonists target the effector cells producing inflammatory cytokines, which in turn regulate adaptive and innate mechanisms of inflammation (Ward-Kavanagh et al., 2016). There are also ongoing clinical trials with a BTLA agonist mAb for use in systemic autoimmune diseases (LY3361237, Lilly; National Library of Medicine, 2022b; NCT05123586) in addition to the development of a BTLA selective mutein of HVEM as an Ig fusion protein (AVTX008; Šedý et al., 2017).

Lastly, the LIGHT–HVEM–BTLA pathway is under investigation as a source of therapeutic targets for cancer immunotherapy. Several studies in mouse cancer models indicate LIGHT engineered as soluble and stable agonist enhances cancer immunotherapy (Yu et al., 2004; Skeate et al., 2020). Various strategies are being employed to deliver LIGHT into the tumor microenvironment including viral vectors (Jazowiecka-Rakus et al., 2021) and soluble LIGHT tagged with vascular targeting peptide (He et al., 2020). A LIGHT-TIGIT (T cell immunoreceptor with Ig and ITIM domains) fusion molecule is in development to provide both checkpoint inhibition and co-stimulation (SL-9258; Shattuck Labs). However, the pharmacologic hurdles facing the development of soluble multimeric proteins to match those of antibodies are yet to be surmounted. Accumulating evidence also indicates targeting BTLA as a path to enhance immunotherapy (Šedý and Ramezani-Rad, 2019). One idea is based on the redundant use of SHP1/2 phosphatases by both PD1 and BTLA as the mechanism attenuating TCR activation; thus blocking one of these checkpoints leaves the other active (Celis-Gutierrez et al., 2019). PD1 and BTLA checkpoints regulate different aspects of the T cell immune response, suggesting a combination approach with their respective inhibitors may be effective in overcoming PD1 resistance. A newly launched clinical trial (National Library of Medicine, 2019; NCT04137900) will investigate an inhibitory mAb to BTLA in combination with an anti-PD1 mAb to test this hypothesis.

## Conclusion

The advances in understanding the molecular and physiologic functions of the TNFSF members have resulted in an improved perspective of the complex functions of the LIGHT network in innate and adaptive immune responses. Additionally, the characterization of genetic and other regulatory mechanisms that may affect LIGHT signaling networks have revealed new strategies to treat a wide range of diseases, including autoimmune disorders, inflammatory diseases, and cancer. Results in clinical trials with antagonists have demonstrated the potential role of LIGHT in some inflammatory conditions, including the impact of LIGHT neutralizing mAb in the pathogenesis of COVID-19. Collectively, targeting LIGHT could yield a novel therapeutic opportunity for treating immune-related pathologies.
